# Dysbiosis and reduced small intestinal function are required to induce intestinal insufficiency in mice

**DOI:** 10.1152/ajpgi.00201.2022

**Published:** 2022-11-08

**Authors:** Peggy Berlin, Israel Barrantes, Johannes Reiner, Emma Schröder, Brigitte Vollmar, Jana Bull, Bernd Kreikemeyer, Georg Lamprecht, Maria Witte

**Affiliations:** ^1^Division of Gastroenterology, Department of Medicine II, Rostock University Medical Center, Rostock, Germany; ^2^Institute for Biostatistics and Informatics in Medicine and Ageing Research, Rostock University Medical Center, Rostock, Germany; ^3^Rudolf-Zenker-Institute for Experimental Surgery, Rostock University Medical Center, Rostock, Germany; ^4^Institute for Microbiology, Virology and Hygiene, University Medicine Rostock, Rostock, Germany; ^5^Department of General, Visceral, Thoracic, Vascular and Transplant Surgery, Rostock University Medical Center, Rostock, Germany

**Keywords:** cecum, gut microbiota, ileocecal valve, intestinal resection, short bowel syndrome

## Abstract

Extensive bowel resection can lead to short bowel syndrome and intestinal failure. Resection-induced dysbiosis may be related to the specific anatomic site of resection and influences the disease progression. Although patients with end-jejunostomy are at high risk for intestinal failure, preservation of the ileocecal valve and colon counteracts this risk. The present study investigated the role of the cecum in maintaining microbial homeostasis after different types of small bowel resection. Male C57BL6/J mice were anesthetized by intraperitoneal injection of ketamine-xylazine and received extended ileocecal resection (extended ICR), limited ileocecal resection (limited ICR), or mid-small bowel resection (SBR). Stool samples were collected before surgery and between *postoperative days 2–7*, for 16S rRNA gene sequencing. Only extended ICR, but neither limited ICR nor SBR, induced intestinal insufficiency. α-Diversity was reduced in both ICR variants but not after SBR. All resections resulted in an increase in Proteobacteria. Pathobionts, such as *Clostridia*, *Shigella*, and *Enterococcus*, increased after SBR while *Muribaculaceae*, *Lactobacillus*, and *Lachnospiraceae* decreased. Limited ICR resulted in an increase of members of the *Clostridium sensu stricto group*, *Terrisporobacter* and *Enterococcus* and a decrease of *Muribaculaceae*. The increase of *Enterococcus* was even more pronounced after extended ICR while *Muribaculaceae* and *Akkermansia* were dramatically reduced. Both ICR variants caused a decrease in steroid biosynthesis and glycosaminoglycan degradation-associated pathways, suggesting altered bile acid transformation and mucus utilization.

**NEW & NOTEWORTHY** Resection-induced dysbiosis affects disease progression in patients with short bowel syndrome. Severe dysbiosis occurs after removal of the ileocecal valve, even in the absence of short bowel conditions, and is associated with the loss of *Muribaculaceae* and *Akkermansia* and an increase of *Clostridium* and *Enterococcus.* The preservation of the cecum should be considered in surgical therapy, and dysbiosis should be targeted based on its specific anatomical signature to improve postoperative bacterial colonization.

## Graphical abstract

**Figure:**
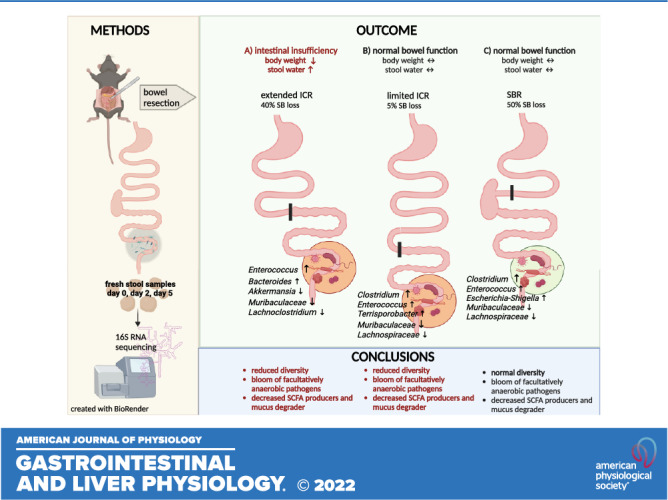

## INTRODUCTION

Intestinal resection is a frequent intervention in adult and pediatric patients with Crohn’s disease and other gastrointestinal diseases (1). Extended or multiple resections may lead to short bowel syndrome (SBS) and intestinal failure (IF). SBS describes a condition of intestinal insufficiency that is associated with malabsorption of macronutrients and/or water and electrolytes. Patients with IF require intravenous supplementation to maintain health and/or growth (2). Although parenteral nutrition is lifesaving in IF, long-term application is often associated with severe complications such as liver disease and bloodstream infections (3). Because of the development of dilated loops and reduced peristalsis small intestinal bacterial overgrowth (SIBO) may occur (4). Subsequently, SIBO increases SBS-associated morbidity and liver complications by attributing to bacterial translocation and mucosal inflammation, and sometimes d-lactic acidosis (4). A correlation between SBS severity and microbial changes has also been suggested (5).

The severity and prognosis of SBS strongly depend on the postoperative anatomy (6). Patients with SB can be categorized into three different groups with distinct severity and disease prognosis: type I: end-jejunostomy; type II: jejunocolic anastomosis; and type III: jejuno-ileal anastomosis. Type I patients have the lowest chance of successful intestinal adaptation and are at high risk to develop chronic IF, whereas type III patients, having the ileocecal valve and the colon in continuity, have lesser fluid losses and are at low risk for permanent IF (7, 8). Changes in the intestinal microbiome also depend on SB anatomy. The ileocecal junction slows the intestinal transit, allows for bacterial fermentation, and prevents the reflux of luminal contents from the large into the small intestine. A prospective study by Huang et al. (9) revealed that the stool microbiota of type II patients lacking the ileocecal junction differed from the composition in type III patients and that bacterial α-diversity positively correlated with the length of the remnant small bowel in these patients. Similar findings were obtained from stool analysis in rats after 75% ileocecal resection when compared with small bowel-only resected rats (10). However, in addition to the site of anastomosis, the etiology of SBS and medical treatment (SIBO prophylaxis) may also have a strong impact on microbial colonization. Thus, the interpretation of microbial changes after intestinal resection in patients is somewhat difficult and experimental models are needed to shed light on the site-specific changes in the microbiome.

In this study, we not only tested the effects of an absent ileocecal valve on the stool microbiota but also considered different extents of ileocecal resection in mice. Our data show that limited resection of the cecum only, leaving most of the ileum intact and the colon in continuity, is sufficient to induce severe structural and functional disturbances of the colonic microbiota. In contrast, loss of a comparable bowel length after mid-small bowel resection induced only slight changes in microbial composition. This finding suggests that the loss of the cecum causes intestinal dysbiosis in mice, even in the absence of short bowel conditions leading to malabsorption and diarrhea.

## MATERIALS AND METHODS

### Ethical Considerations

All animal experiments were performed according to the EU Directive 2010/63/EU of the animal protection law and were approved by the local governmental administrations (Landesamt für Landwirtschaft, Lebensmittelsicherheit und Fischerei Mecklenburg-Vorpommern, 7221.3-1.1–067/19 and 7221.3-1.1–034/20).

### Surgical Intervention

**Figure 1.**
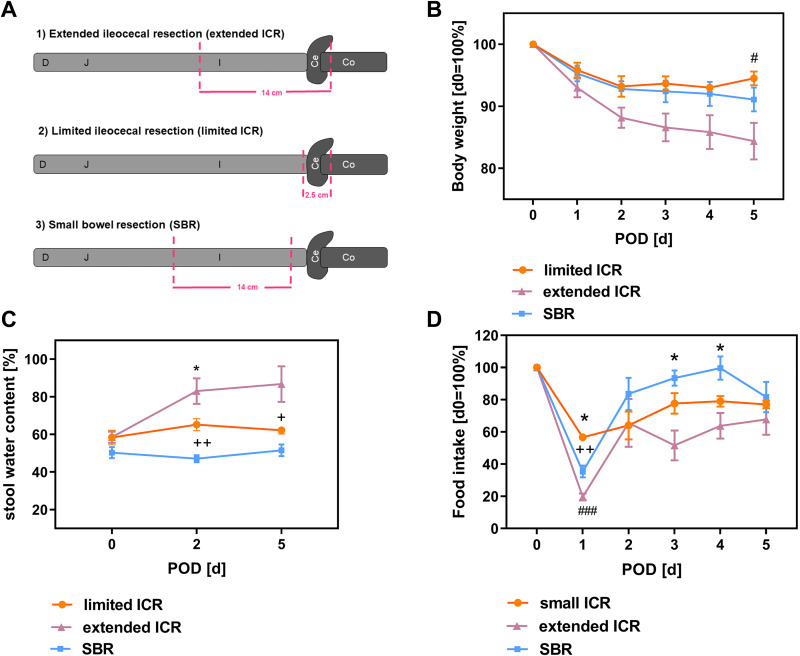
*A*: scheme of bowel resection variants performed in mice. Male mice underwent either *1*) extended ileocecal resection (extended ICR) with removal of the ileocecal junction and the whole ileum, *2*) limited ileocecal resection (limited ICR) with removal of the ileocecal junction, or *3*) 50% small bowel resection (SBR) leaving the ileocecal junction intact. Body weight (*B*), stool water content (*C*), and food intake (*D*) from mice with limited ileocecal resection (limited ICR, *n* = 5 or 6), extended ileocecal resection (extended ICR, *n* = 5 or 6), and isolated small bowel resection (SBR, *n* = 6–10) as percentage. Values are given as means ± SE. Two-way ANOVA was used to compare mean differences between the groups. Tukey’s test was involved to correct for multiple comparisons. *B*: *postoperative day* (POD) *5* #*P*_adjust_.= 0.039 limited ICR vs. extended ICR. *C*: *postoperative day* (POD) *2* *P_adjust_. = 0.021 extended ICR vs. SBR, ++*P*_adjust_.= 0.0028 (limited ICR vs. SBR). *POD 5* +*P*_adjust_. = 0.039 limited ICR vs. SBR. *D*: *postoperative day* (POD) 1 ++*P*_adjust._= 0.006 limited ICR vs. SBR, ###*P*_adjust._ < 0.0001 limited ICR vs. extended ICR, *P_adjust_. = 0.02 extended vs. SBR. *POD 3* *P_adjust._ = 0.016 extended vs. SBR, *POD 4* *P_adjust_. = 0.022 extended vs. SBR. Ce, cecum; Co, colon; D, duodenum; I, ileum; J, jejunum.

**Figure 2.**
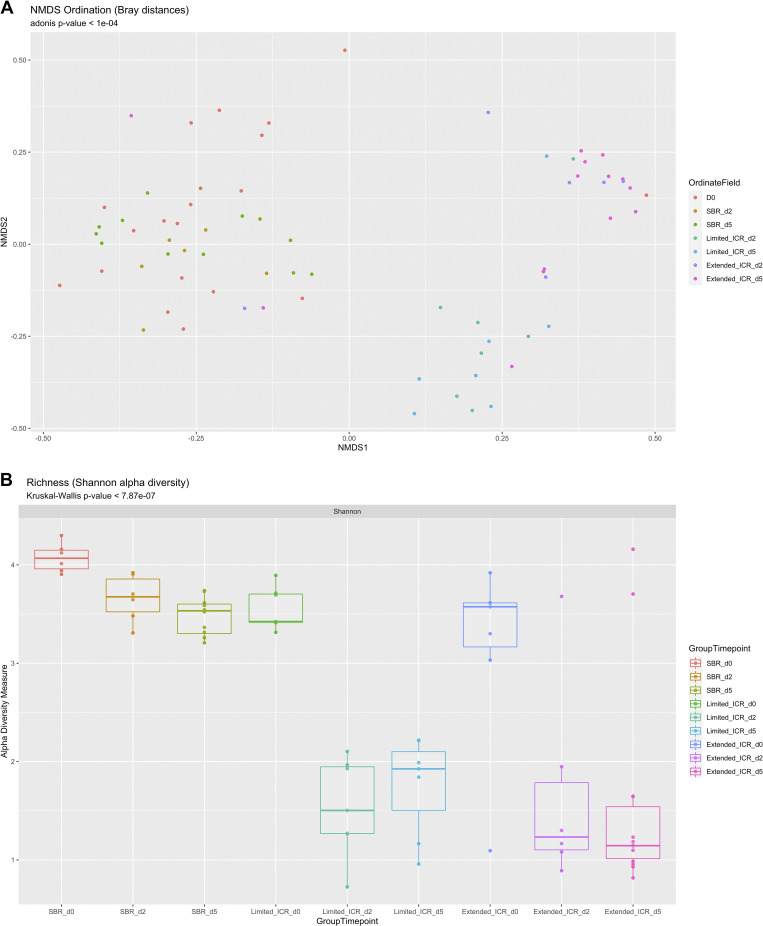
Nonmetric multidimensional scaling (NMDS) ordination analysis (*A*) and Shannon α-diversity of the bacterial communities (*B*) in the stool of male mice before surgery (d0, baseline, SBR, *n* = 6, limited ICR, *n* = 7, extended ICR, *n* = 6), at *postoperative day* (POD) 2 and *POD 5* after small bowel resection (SBR_d2, *n* = 6 and 5, *n* = 12), after limited ileocecal resection (Limited_ICR_d2, *n* = 7 and d5, *n* = 7), and after extended ileocecal resection (Extended_ICR_d2, *n* = 6 and d5, *n* = 12). *A*: each dot represents one stool sample, *P* < 0.0001, PERMANOVA. *B*: values are given as box and whiskers (min to max). Groups were compared using the Kruskal–Wallis test, *P* = 7.87 × 10^7^.

**Figure 3.**
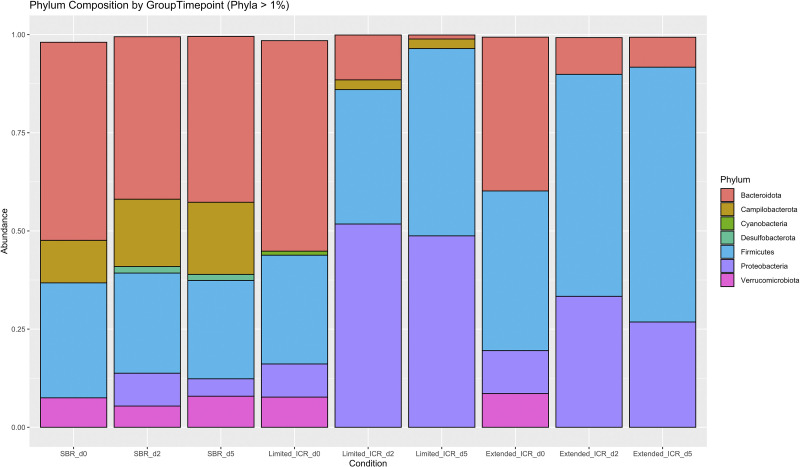
Phylum composition of bacterial communities (>1%) in the stool of male mice before surgery (d0, baseline SBR, *n* = 6, limited ICR, *n* = 7, extended ICR, *n* = 6), at *postoperative day* (*POD*) *2* and *POD 5* after small bowel resection (SBR_d2, *n* = 6 and 5, *n* = 12), after limited ileocecal resection (Limited_ICR_d2, *n* = 7 and d5, *n* = 7), and after extended ileocecal resection (Extended_ICR_d2, *n* = 6 and d5, *n* = 12).

C57BL6/J mice were bred in the animal facility under specific pathogen-free (SPF) conditions at the Rudolf-Zenker-Institute of Experimental Surgery, University Medical Center Rostock. Because the experimental short bowel model was originally established in male mice in our laboratory and to exclude possible sex-specific variations in modulation gut microbiota in the overall small sample, only male mice were used in this study. Mice were housed under conventional conditions with free access to standard chow and water. Mice were used for experiments at the age of 3–6 mo. Two days before surgery mice were separated into single cages and switched to a standard liquid diet (AIN 93 G, ssniff, Soest, Germany) until the end of the experiment to prevent postoperative intestinal obstruction. As previously shown, a liquid diet has no influence on the microbial composition (11). Mice underwent 50% small bowel resection (SBR, *n* = 10 animals) leaving the ileocecal junction intact, limited ileocecal resection (limited ICR, *n* = 7 animals) with removal of the ileocecal valve only, or extended ileocecal resection (extended ICR, *n* = 6 animals) with removal of the ileocecal valve and the entire ileum as outlined in Fig. 1. In detail, mice were weighted and anesthetized by intraperitoneal injection of ketamine and xylazine. To prevent intraoperative hypoxia mice were orally intubated with a 22-gauge cannula and mechanically ventilated with room air (200 µL and 120 breaths/min) using a mouse ventilator (Minivent 845, Hugo-Sachs Elektronik-Harvard Apparatus GmbH, March-Hugstetten, Germany). Mice were placed on a heating plate and body temperature was continuously monitored. The operative technique of ileocecal resection was performed as previously described (11). After midline incision was made, the ileocecal junction and the ileum were eviscerated. In mice undergoing extended ICR the mesenteric blood vessels of the resected segment were ligated using clips (Weck, Horizon, Teleflex Medical, Kernen, Germany) and the intestine was divided 12 cm proximal to the ileocecal valve and immediately distal to the cecum (Fig. 1*A*, *1*). The ileum and the cecum (∼14 cm) were resected, and intestinal continuity was restored by an end-to-end jejunocolic anastomosis with 11–13 interrupted stitches using a 10-0 monofilament suture (Ethilon, Ethicon, Norderstedt, Germany). For the limited ICR, the ileum was divided ∼1 cm proximal to the ileocecal valve and immediately distal to the cecum. The anastomosis was created in the same way as described earlier (Fig. 1*A*, *2*). For the mid-small bowel resection (SBR) the jejunum was transected ∼16 cm and 2 cm proximal to the ileocecal valve and the intermediate 14 cm long small intestinal segment was resected. Continuity was restored by a small bowel end-to-end jejuno-ileo anastomosis in the same way as described for ICR (Fig. 1*A*, *3*). During the resections, specific attention was paid not to cause ischemia at the ends of the intestinal segments that were put together by the anastomosis. Before closure, the abdomen was irrigated with 1 mL saline to prevent adhesions. Immediately after extubation mice were weighted and resuscitated with subcutaneous injection of 1 mL Sterofundin and 5 mg/kg body wt (BW) carprofen as an analgesic. Mice were allowed to recover in a heated terrarium (29°C) and then returned to cages with free access to liquid food and metamizole containing (1.25 mg/mL, 200 mg/kg BW) water. Mice were killed on *day 5* (limited ICR) or *day 7* (SBR, extended ICR) under deep isoflurane anesthesia by cervical dislocation.

### Clinical Examination and Humane Endpoints

Body weight, food intake, and wellness score of mice were recorded daily until *day 5* as described previously (11). Wellness score was used to assess the general condition of mice. Eleven points correspond to maximal well-being. A wellness score below 4 points, a distended abdomen with coprostasis suggestive of postoperative ileus or massive diarrhea combined with dehydration indicating decompensated short bowel were defined as humane endpoints, and mice were killed immediately.

### Measurement of Stool Water Content

Stool water content was measured on the day of surgery (0d) and on the *postoperative days* (POD) *2* and *5* as previously described (11). In brief, fresh stool was collected in preweighed tubes [*e*], which were immediately closed. After weighing again [*w*], tubes with fresh stool were opened and placed in a hot air oven at 85°C. After 24 h, the tube was once again weighed [*d*], and stool water content (%) was calculated as follows: (*w* − *d*)/(*w* − *e*) × 100.

### Stool Sample Collection and DNA Extraction

Fresh stool samples were collected on the day before surgery (*POD 0*) at *POD 2* and at *POD 5*. For mice with SBR and extended ICR, stool samples were additionally collected on *day 7*, which were pooled with the samples on *day 5* for further analysis and designated as *POD 5*. Stool was flash-frozen and immediately stored at −80°C until analyzed. Total DNA was extracted using ZymoBIOMICSTM DNA Miniprep Kit according to the manufacturer’s protocol (ZYMO RESEARCH, Freiburg, Germany) including bead-beating system to ensure unbiased lysis of bacteria.

### Preparation of 16S rRNA Gene Sequencing Libraries and Sequencing

The concentration of purified DNA was determined by Qubit and Nanodrop protocols and finally the amplicon PCR was started with DNA templates at a concentration of 5 ng/µL in 10 mM Tris, pH 8.5. The bacterial 16S rRNA genes were amplified using 515 F (5′-TCG-TCG-GCA-GCG-TCA-GAT-GTG-TAT-AAG-AGA-CAG-GTG-CCA-GCM-GCC-GCG-GTA-A-3′) and 806 R (5′-GTC-TCG-TGG-GCT-CGG-AGA-TGT-GTA-TAA-GAG-ACA-GGG-ACT-ACH-VGG-GTW-TCT-AAT-3′) primers targeting the V4 region. The PCR resulted in amplicon sizes of ∼390 bp. All further steps in library preparation were performed according to the Illumina “16S Metagenomic Sequencing Library Preparation” protocol and as described previously (12). Briefly, PCR clean-up, Index PCR, PCR clean-up 2, library quantification, normalization, and pooling were performed according to the above-referred manual. Bioanalyzer DNA 1000 chips (Agilent Technologies) and Qubit kits (Thermo Fischer Scientific) were used for quantity and quality controls of each individual sample library and the final library pool. Ten percent PhiX control was spiked into the final pool. 5 pM of the final library pool was subjected to one individual sequencing run using a 600-cycle V3 chemistry kit on an Illumina MiSeq machine. During the run, roughly 1,100 (k/mm^2^) clusters were sequenced, generating ∼25 million reads passing filter specs. Over 69.0% of the sequencing and index reads were found with a *Q*_score_ ≥ 30.

### Microbiome Bioinformatics

16S Amplicons were merged from the paired-end Illumina sequencing runs using VSEARCH (13). Merged fragments were then denoised with deblur (14), and operational taxonomic units (OTUs) were assigned to the denoised fragments with the sklearn classifier (15) against 16S rRNAs clustered to 99% identity from the SILVA database version 138 (16). Both deblur and sklearn were used within the QIIME2 pipeline version 2020.11 (17). The obtained OTU table was then processed with phyloseq (18), a bioconductor R package to evaluate the ordination, abundance, and composition of the bacterial communities in the samples (19). Statistical significance of these analyses was assessed through the permanova (adonis) and Kruskal–Wallis tests, as implemented in the vegan package (20). Besides, significant statistical differences between the genera abundances pertaining to the different sample groups were calculated with the DESeq2 R package (21), applying a Wald’s test and the Benjamin–Hochberg correction for the *P* values (false discovery rate cutoff: FDR < 0.01). The obtained differentially abundant OTUs were then binned into time-series groups via the fuzzy clustering method implemented in the Mfuzz package version 2.38.0 (22). In addition, to observe the genera shared between the different communities we followed the protocol developed in the microbiome R package (version 1.9.96) to calculate the core microbiomes for each sample group (23, 24). Furthermore, we summarized the OTUs by bacterial phenotypes into each sample group, via the BugBase server (accessed 2021-06-20) (25) To this end, we reassigned OTUs against the Greengenes 16S database version 13.5 (26), as this is the latest version compatible with BugBase. Then, a PICRUSt analysis was carried out to predict the relative abundance of functional pathways in the microbial communities (27). Here, we also employed the OTUs assigned against Greengenes 13.5 and obtained OTU tables were then normalized by 16S rRNA copy number to predict functional genes from the Kyoto Encyclopedia of Genes and Genomes (KEGG) database (28). Finally, the results from the PICRUSt analysis and KEGG predictions were processed with the phyloseq R package as well.

### Statistic of Clinical Data

Body weight, stool water content, and food intake were analyzed using GraphPad Prism 9.0 (GraphPad Software). Data are presented as means ± standard error (SE) for the indicated number of mice (*n*) per group. Group comparisons were performed by two-way ANOVA. Tukey’s test was used to correct for multiple comparisons. Significance level of 0.05 or adjusted α (α_adjust_) as indicated in the figure legends was considered.

## RESULTS

### Removal of the Ileocecal Valve Is Sufficient to Induce Stool Water Increase but Not to Induce Intestinal Insufficiency in Mice

Mice underwent extended ICR, limited ICR, or SBR as shown in Fig. 1*A*. Body weight was significantly decreased in mice with extended ICR (extended ICR vs. limited ICR *P*_adjust._ = 0.039, Fig. 1*B*). Stool water content was significantly elevated at *POD 2* after limited ICR (limited ICR vs. SBR *P*_adjust_. = 0.003, Fig. 1*C*) and even more increased after extended ICR (extended ICR vs. SBR *P*_adjust_. = 0.02, Fig. 1*C*). At later time points, fecal water content remained elevated after ICR although this was not statistically significantly for mice with extended resection (limited ICR vs. SBR *P*_adjust._ = 0.039, extended ICR vs. SBR *P*_adjust._ = 0.058, Fig. 1*C*). Food intake was transiently decreased on the first day after resection in all groups but recovered beyond *POD 2* (Fig. 1*D*). In contrast, food intake remained significantly decreased after extended ICR compared with small bowel resected mice (extended ICR vs. SBR, *P*_adjust._ < 0.05 *POD 3*, *POD 4*, Fig. 1*D*). None of the mice became hyperphagic.

### Removal of the Cecum Induces Decreased α-Diversity and Disturbances of the Colonic Microbial Composition, Even in the Absence of Intestinal Insufficiency

Stool samples were analyzed by 16S rRNA sequencing on the day of surgery (d0, baseline) at *POD 2* and at *POD 5*. Based on multidimensional scaling microbial composition of stool samples was not different between the groups before resection (Fig. 2*A*, red dots). Stool samples obtained from mice on *POD 2* and *POD 5* after SBR did not separate from baseline. In contrast, stool samples taken from mice after limited ICR and after extended ICR were separated into different clusters (Fig. 2*A*). Shannon α-diversity of stool samples taken before surgery (baseline) did not differ between the groups. In line with the multidimensional scaling analysis, α-diversity remained stable after SBR. In contrast, at *POD 2* after limited ICR α-diversity dramatically decreased compared with baseline and remained decreased at *POD 5*. The same was true for the stool samples of mice obtained after extended ICR. Here, Shannon α-diversity decreased even further (Fig. 2*B*).

To describe the general characteristics of microbiota after the three different resection variants, we analyzed the composition of stool microbiota (Fig. 3). Before surgery (0d) phylum composition of bacterial communities in the stool was similar between the groups. Bacteroidota was the most abundant phylum followed by Firmicutes and Verrucomicrobiota. In addition, Campilobacterota, Cyanobacteria, and Desulfobacterota were also detected at low levels in the stool of mice before surgery. After surgery, phylum composition changed within the groups and became different between the groups. After SBR, there was no major impact on the most abundant phyla, Bacteroidota and Firmicutes, but a slight increase in Desulfobacterota and Proteobacteria. In contrast, both after limited and after extended ICR there was an overall increase of Firmicutes and an overall decrease of Bacteroidota, and Proteobacteria became more abundant. In addition, after limited ICR only, there also was a slight increase of Campilobacterota. Of note, after limited and extended ICR the abundance of Verrucomicrobiota decreased below 1%. Taken together, the presence of the cecum is necessary to maintain the general composition of the colonic microbial community independent of the length of the resected small bowel.

**Figure 4.**
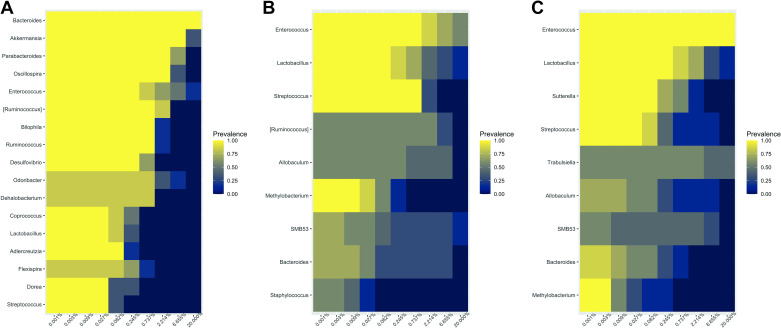
Comparison of the 20% most abundant groups of microbiota in the feces of male mice at *postoperative day* (POD) *5* after small bowel resection (SBR, *n* = 12; *A*), limited ileocecal resection (limited ICR, *n* = 7; *B*), or extended ileocecal resection (extended ICR, *n* = 12; *C*).

Consistent with the findings at phylum level, comparison of the core taxa (20% relative abundance) at *POD 5* revealed notable differences among the three resection variants (Fig. 4). Five days after SBR, the core microbiome of mice was diverse with Bacteroides being the most abundant genus followed by Akkermansia (Fig. 4*A*). After removal of the cecum, the core microbiome impoverished independent of the extent of resection. In mice that underwent limited ICR (Fig. 4*B*) and also in mice with short bowel conditions after extended ICR (Fig. 4*C*), gram-positive aerotolerant bacteria *Enterococcus*, *Lactobacillus*, and *Streptococcus* had the highest prevalence within the core microbiome of the host.

**Figure 5.**
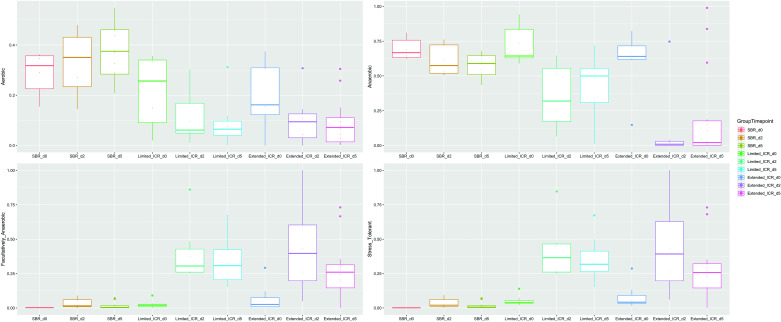
Proportion of aerobic, anaerobic, facultative anaerobic, and stress tolerant bacteria in the stool of male mice after small bowel resection (SBR d2, *n* = 6 and 5, *n* = 12), limited ileocecal resection (limited ICR; d2, *n* = 7 and d5, *n* = 7), and extended ileocecal resection (extended ICR d2, *n* = 6 and d5, *n* = 12) before surgery (d0, baseline, SBR, *n* = 6; limited ICR, *n* = 7; extended ICR, *n* = 6) and at *postoperative day* (*POD*) *2* and *POD 5* after resection. Values are given as box and whiskers (min to max).

**Figure 6.**
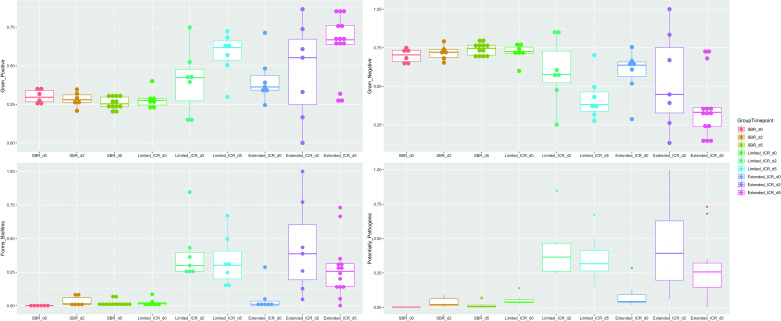
Proportion of gram-positive, gram-negative, biofilm-forming, and potentially pathogenic bacteria in the stool of male mice after small bowel resection (SBR d2, *n* = 6 and d5, *n* = 12), limited ileocecal resection (limited ICR d2, *n* = 7 and d5, *n* = 7), and extended ileocecal resection (extended ICR d2, *n* = 6 and d5, *n* = 12) before surgery (d0, baseline, SBR, *n* = 6; limited ICR, *n* = 7; extended ICR, *n* = 6), and at *postoperative day* (*POD*) *2* and *POD 5* after resection. Values are given as box and whiskers (min to max).

Under physiological conditions, the colon habitats a diverse community of bacteria that are mainly anaerobic. The number of aerobic and anaerobic bacteria was stable in the feces of mice after SBR (Fig. 5). In contrast, ileocecal-resected mice mostly harbor aerobic and anaerobic bacteria and a great amount of facultative anaerobe, stress-tolerant bacteria, in the colon. Moreover, these mice display an increasing shift from gram-negative to gram-positive bacteria, which was most evident after extended ICR, and a dominant growth of biofilm-forming and potentially pathogenic species (Fig. 6).

### Small Bowel and Ileocecal Resection Reveals Distinct Patterns of Differentially Abundant Taxa

**Figure 7.**
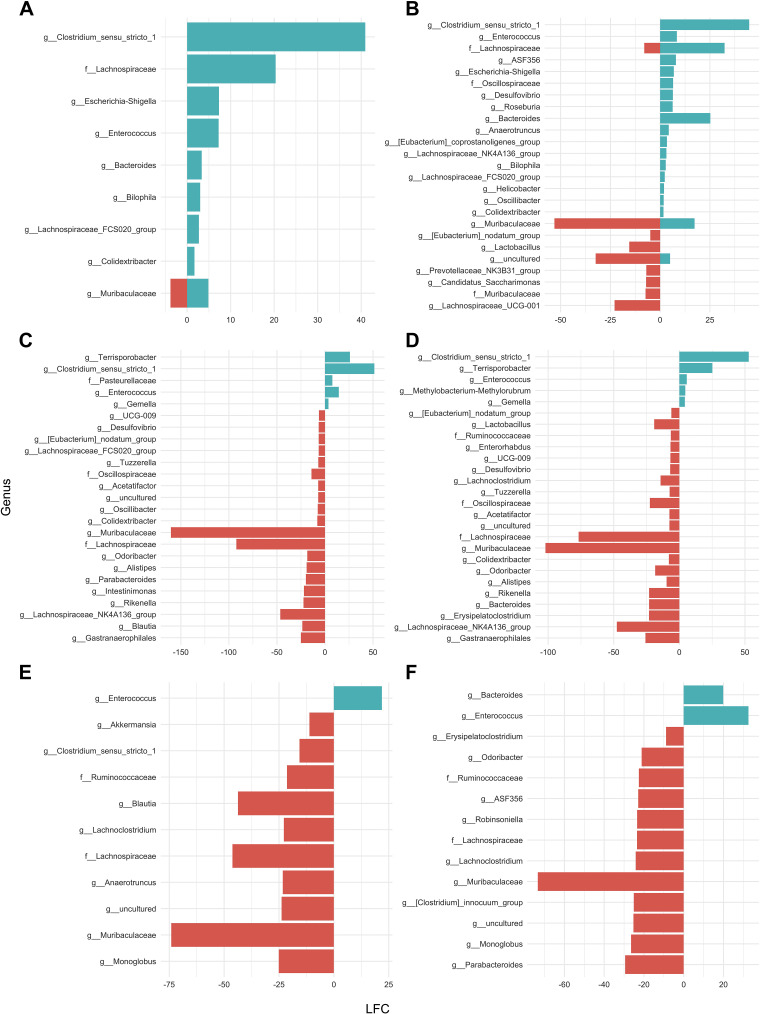
Differential abundance of taxa through time. The significant changes in the male mice with small bowel resection (SBR, *n* = 6–12; *A* and *B*); those with the limited ileocecal resection (ICR, *n* = 7 per time point; *C* and *D*); and with the extended ICR (*n* = 6–12; *E* and *F*); in all cases at 2 and 5 days, respectively. The *x*-axis scale is in log2-fold-changes (LFC), and genera are listed in the *y*-axis. Family instead of the genera names were included when there was no description at the genus level. Blue bars indicate increase in fold changes (LFCs > 0), and red ones the opposite. Significant statistical differences between the genera abundances pertaining to the different sample groups were calculated with the DESeq2 R package (21) applying a Wald’s test and the Benjamin–Hochberg correction for the *P* values (false discovery rate cutoff: FDR < 0.01).

The composition of the stool microbiota was checked for significant changes at the genus level. The log2-fold-changes of differentially abundant OTUs on *POD 2* and *POD 5* are shown in Fig. 7. At *POD 2* after SBR, most differentially abundant OTUs were increased and belonged to the phyla Firmicutes and Bacteroidota or Proteobacteria. At genus level, *Clostridium senso stricto 1* (log2-fold-change 40), *Echericha-Shigella* (log2-fold-change 7), and *Enterococcus* (log2-fold-change 7) were increased on *POD 2* after SBR (Fig. 7*A*) and remained significantly increased on *POD 5*. OTUs belonging to *Bacteroides* and to some members of *Muricabulaceae* were significantly increased on *POD 5* after SBR (Fig. 7*B*).

In contrast to the changes detected after SBR, differential abundant OTUs were mainly decreased after limited ICR and only a few were elevated. Namely, OTUs belonging to *Muribaculaceae* (Bacteriodetes) were dramatically reduced on *POD 2* and *POD 5* after limited ICR (log2-fold-change −160 on *POD 2*, log2-fold-change −100 on *POD 5*). In addition, OTUs belonging to the *Lachnospiraceae_NK4A 136* group, *Rikinella, Lactobacillus*, and *Bacteroides* were also decreased after limited ICR. After limited ICR *Clostridium sensu stricto 1* (Firmicutes) again was significantly increased (log2-fold-change 50) on *POD 2* and *POD 5*. In addition to the appearance of *Enterococcus*, *Terrisporobacter* (both Firmicutes) significantly increased (log2-fold-change 25) on *POD 2* and *POD 5* after limited ICR (Fig. 7, *C*and *D*).

Similar to limited ICR, differential abundant OTUs were mainly decreased after extended ICR, and only a few (*Enterococcus*, *Bacteroides*) were increased. OTUs belonging to *Muribaculaceae* were strongly decreased (log2-fold-change −70 on *POD 2* and *POD 5*) after extended ICR. In addition, extended ICR also significantly decreased the abundance of *Akkermansia* on *POD 2* (log2-fold-change −11, Fig. 7*E*). In contrast to SBR and limited ICR, the stool of mice after extended ICR revealed significantly decreased OTUs of *Clostridium sensu stricto 1* at *POD 2* (log2-fold-change −15, Fig. 7*E*). In addition, the abundance of *Enterococcus* was even more increased at both time points after extended ICR than after SBR and limited ICR (Fig. 7, *E*and *F*).

### Small Bowel and Ileocecal Resection Induces Distinct Genera Abundance Dynamics in the Colon Microbiome of Mice

**Figure 8.**
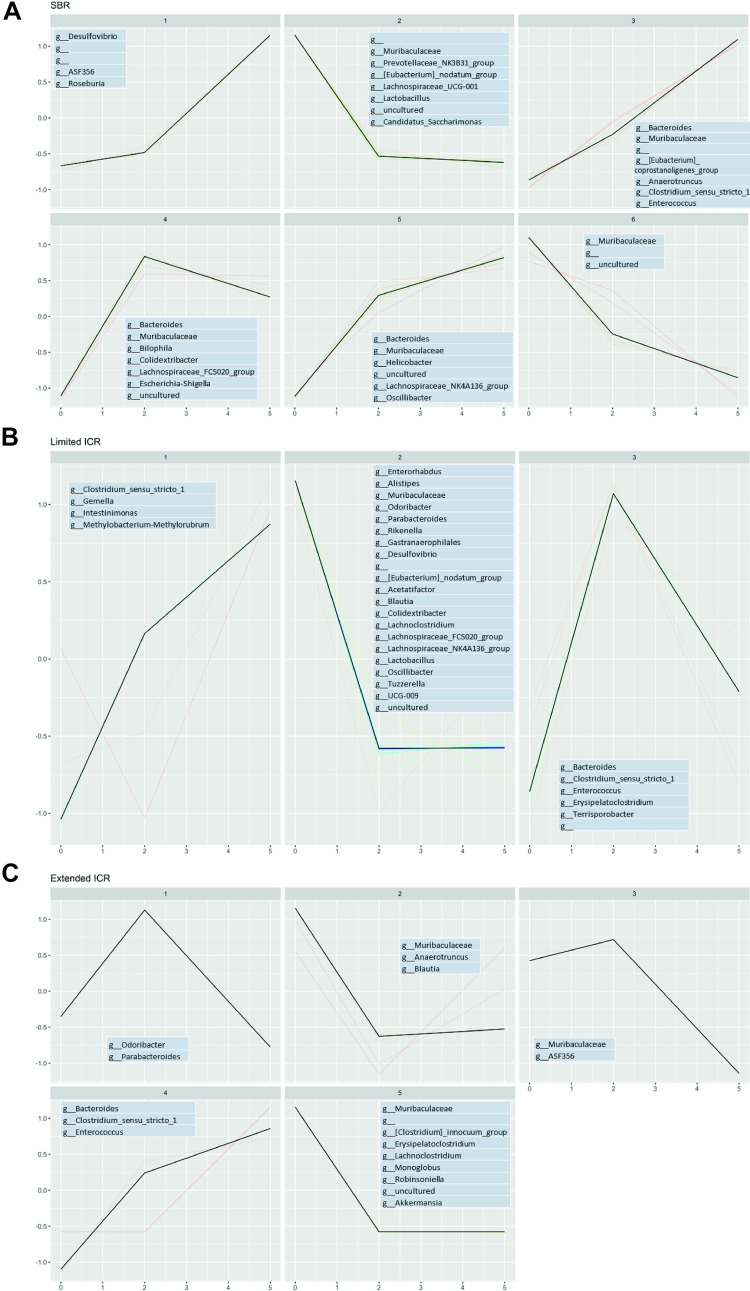
The *x*-axis represents the time dynamics (*postoperative days 0*, *2*, and *5*), whereas the *y*-axis corresponds to the genera abundance changes in the feces of male mice after small bowel resection (SBR, *n* = 6–12 per time point; *A*), limited ileocecal resection (limited ICR, *n* = 7 per time point; *B*), and after extended ileocecal resection (extended ICR, *n* = 6–12 per time point; *C*). These abundance changes were calculated from the DESeq2-normalized OTU abundances for each genera, which were then standardized to have a mean value of zero and a standard deviation of one for each OTU abundance profile. Afterward, these transformed OTU abundances were clustered using the fuzzy c-means clustering algorithm implemented in Mfuzz (22), and their transformed abundances plotted against time. OTUs with a cluster membership value ≥ 0.90 are shown as black lines. OTUs, operational taxonomic units.

To emphasize the dynamics of the observed effects, abundance changes were calculated, and transformed OTUs were clustered. For the SBR samples, we identified six clusters. Clusters consisted of taxa whose relative abundance first increased fast, and then either abundance kept increasing but in slower increments (*cluster C5*) or decreased (*cluster C4*). Conversely, we also found clusters in the SBR samples whose abundance increased slowly until *POD 2*, and then either kept increasing but at a faster pace (*cluster C1*) or increased fast until *POD 2* and then increased even faster (*cluster C3*). Finally, we observed two clusters, which include taxa whose abundances decreased fast until *POD 2* and then either decreased further slowly (*cluster C6*) or stayed at similar abundances (*cluster C2*; Fig. 8*A*).

Of note, for the limited ICR samples, we observed only three kinds of abundance dynamics: One, increasing until *POD 2*, then decreasing (*cluster C3*); another also increasing until *POD 2* but then keeping increasing (*cluster C1*); and a third, decreasing until *POD 2*, and then remaining stable (*cluster C2*). In contrast, the group of bacteria that decreased until *POD 2* and then remained stable (*cluster C2*) was more diverse and consisted of 126 OTUs (Fig. 8*B*).

Finally, the extended ICR samples exhibited the following dynamics that can be classified into five clusters: Increased abundance until *POD 2* and then decrease (*cluster C1*); moderate increase until *POD 2* and then decrease (*cluster C3*); increased abundances until *POD 2* and then kept increasing although with lower fold-changes (*cluster C4*); decreased abundances until *POD 2* and then increased moderately (*cluster C2*); or decreased abundance until *POD 2* and then remained stable (*cluster C5*). This last *cluster C5* was more diverse than *cluster C1–C4* (Fig. 8*C*).

### Signatures of Microbial Functional Pathways Differ after Limited and Extended Ileocecal Resection

**Figure 9.**
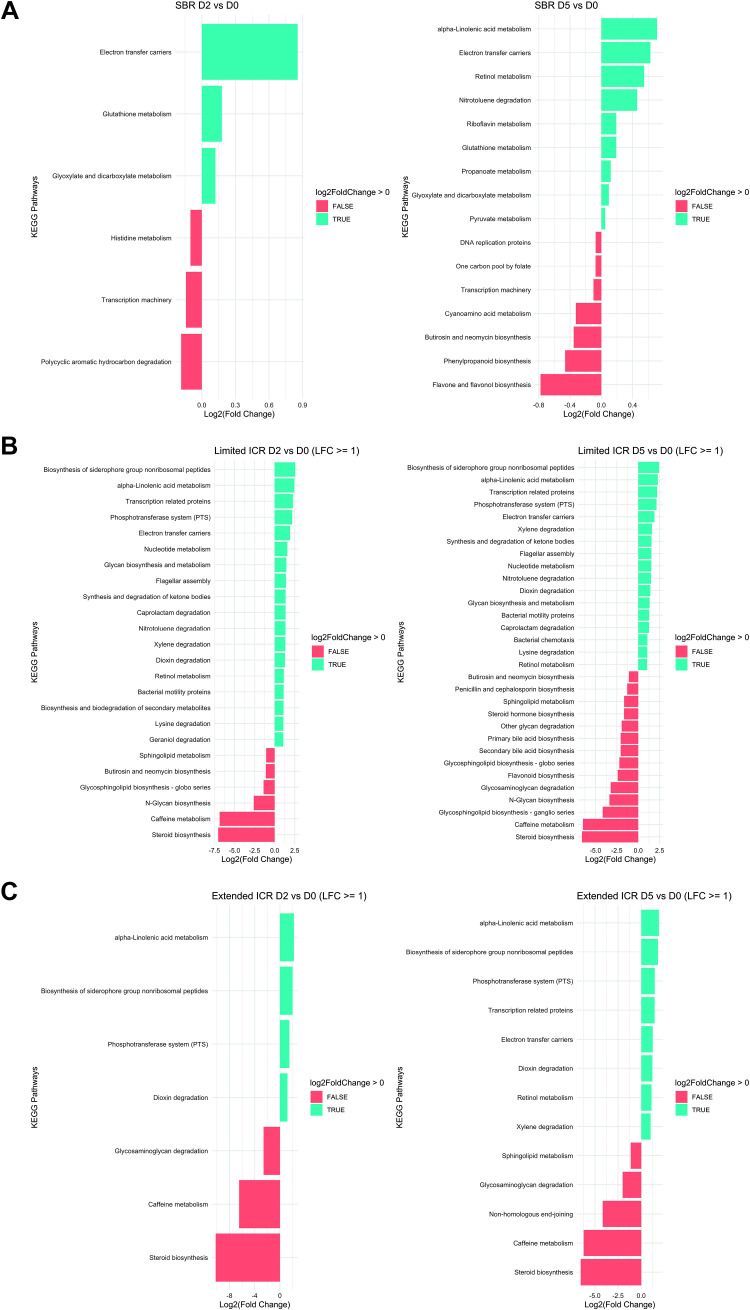
Functional metagenomics pathways of microbiota in the stool of male mice before (*day 0*, baseline SBR, *n* = 6; limited ICR, *n* = 7; extended ICR, *n* = 6), at *postoperative day* (*POD*) *2* or *POD* 5 after small bowel resection (SBR d2, *n* = 6 and 5, *n* = 12) (*A*), limited ileocecal resection (limited ICR, d2, *n* = 7 and d5, *n* = 7) (*B*), or extended ileocecal resection (extended ICR d2, *n* = 6 and d5, *n* = 12) (*C*) predicted by phylogenetic investigation of communities by reconstruction of unobserved states (PICRUSt). Results from the PICRUSt analysis and KEGG predictions were processed with the phyloseq R package. A detailed description is provided in *Microbiome Bioinformatics*.

Finally, we examined the metabolic and functional signature of the colonic microbiota predicted by PICRUSt (Phylogenetic Investigation of Communities by Reconstruction of Unobserved States) after the different resection variants. In line with the observation that SBR induced little changes in the colonic microbiome, functional and metabolic pathways were only marginally different on *POD 2* and *POD 5* compared with baseline. There was a slight and transient 0.8- to 0.9-fold increase in electron carrier pathways on *POD 2* and a slight decrease in pathways involved in flavone and flavonol biosynthesis on *POD 5* (Fig. 9*A*).

In contrast, limited ICR induced marked and stable metabolic and functional changes in the colonic microbiota. Biosynthetic pathways of siderophore group nonribosomal peptides were up to 2.5-fold increased on *POD 2* and *POD 5* and α-linolenic acid metabolism and transcription-related proteins were upregulated on *POD 2* and *POD 5*. Pathways related to caffeine metabolism and steroid biosynthesis were up to 7.5-fold decreased after limited ICR (Fig. 9*B*).

Unlike limited ICR with preservation of most of the ileum, extended ICR with removal of the entire ileum caused an overall decrease of metabolic and functional pathways such as steroid biosynthesis (>8-fold), caffeine metabolism (6.5-fold), and glycosaminoglycan degradation (2.5-fold, Fig. 9*C*), which was most clearly seen at *POD 2* (Fig. 9*C*).

## DISCUSSION

Extended ileocecal resection (extended ICR) and small bowel resection (SBR) are short bowel models to study intestinal insufficiency and mucosal adaptation. We have previously shown that extended ICR induces intestinal insufficiency in mice with persisting increased stool water and weight loss (11, 12) that can be improved by pharmacological treatment (29, 30). As expected, limited ICR did not induce intestinal insufficiency in the present study. But both extended and limited ICR caused significant dysbiosis. Such changes in the intestinal microbiome may have a significant impact on functional adaptation and long-term prognosis of short bowel with intestinal insufficiency (31). Of note, SBR (resecting nearly the same length and nearly the same segment of small intestine as extended ICR) induced neither intestinal insufficiency nor loss of diversity. In line with this, reanastomosis of comparably short segments of small bowel but with intact ileocolonic junction usually converts intestinal failure to intestinal insufficiency in humans (32). Reconstruction of an artificial ileocecal valve has been tried experimentally (33), but this type of reconstructive surgery has not been successfully translated into clinical practice. Nevertheless, the findings of the current study provide a rationale to preserve if at all possible, the ileocecal valve even if extended distal small bowel resection is indicated.

Specific changes in the microbial signature have previously been found in adult patients with type II and type III anatomy and also in rats after 75% SBR compared with 75% ICR (9, 10). Similarly, in our study, after ICR but not after SBR Firmicutes increased, whereas Bacteroidetes und Verrucomicrobia decreased. Thus, ICR induces concordant changes in rats, mice, and humans. Our data additionally show that dysbiosis occurs less dynamically after limited ICR because samples from these mice displayed only three different clusters and most of the taxa showed persistently decreased abundances. Isolated removal of the cecum resulted in a loss of Bacteroidota to the same degree as extended ICR. At the same time, Firmicutes increased in both distal resection variants. Another effect of ICR is the decrease of Verrucomicrobia, which in our study appeared as early as 2 days after removal of the cecum and which in rats remained decreased even 28 days after 75% ICR (10). To date, *Akkermansia muciniphila* and, more recently, also *Akkermansia glycaniphila* have been isolated from the human intestine, the latter being found preferentially in nonmammals (34). *A. muciniphila* is widely distributed in the intestines of human and mice where it colonizes the outer mucosal layer, being most abundant in the cecum (35). *A. muciniphila* increases mucus production and accelerates intestinal epithelial regeneration (36). In addition to *Akkermansia*, *Muribaculaceae* and *Parabacteroides* (both belonging to the phylum Bacteroidota) were also decreased after ICR. All three genera are strong mucus degraders (37). In line with the compositional changes, of these bacteria, limited and extended ICR caused a decrease in steroid biosynthesis and glycosaminoglycan degradation-associated signaling pathways, suggesting altered bile acid transformation and mucus utilization. However, mucus quality and function were not investigated in the present study. Therefore, functional experiments are needed to learn more about the biological consequences of specific microbial changes. Another limitation of the study is that we used only male mice to achieve better homogeneity within the small groups. Therefore, the results should be confirmed in a larger cohort using both sexes, because we cannot rule out sex-dependent effects on the composition of gut microbiota (38). Moreover, morphological adaptation in the murine SBS model occurs within the first 2 wk after extended resection (11). Long-term experiments should demonstrate the stability of the resection-induced changes in the gut microbiota and the ability to improve dysbiosis, for example, by probiotics or their metabolites, in future studies (39, 40).

### Conclusions

To the best of our knowledge, this is the first experimental study showing that the removal of the cecum is sufficient to induce severe intestinal dysbiosis in mice but that short bowel conditions are additionally required to induce intestinal insufficiency. Considering that the resected small intestine cannot be restored, dysbiosis should be targeted based on its specific pathophysiological mechanisms.

## DATA AVAILABILITY

The amplicon sequencing data that support the findings of this study are available in the European Nucleotide Archive at https://www.ebi.ac.uk/ena/browser/view/PRJEB52226, Accession No. PRJEB52226. The data that support the findings (of Fig. 1*C*) are available in the Harvard Dataverse at https://doi.org/10.7910/DVN/UHZTIJ.

## GRANTS

This work was supported by a grant from the Deutsche Forschungsgemeinschaft (BE 6292/1-1 to P.B.) and by the FORUN program (889007 to P.B.) as well as the clinician-scientist program (to J.R.) from the Rostock University Medical Center. Further funding was provided through the research project “EnErGie” and by the European Social Fund (ESF), reference: ESF/14-BM-A55-0007/18 (to P.B.), and the Ministry of Education, Science, and Culture of Mecklenburg-Vorpommern. Purchase of the Illumina MiSeq was kindly supported by the EU-EFRE (European Funds for Regional Development) program and funds from the Rostock University Medical Center awarded to B.K.

## DISCLOSURES

No conflicts of interest, financial or otherwise, are declared by the authors.

## AUTHOR CONTRIBUTIONS

P.B. and G.L. conceived and designed research; P.B., E.S., J.B. and M.W. performed experiments; P.B. and I.B. analyzed data; P.B., B.V., B.K., and G.L. interpreted results of experiments; P.B. and I.B. prepared figures; P.B. and I.B. drafted manuscript; I.B., J.R., G.L., and M.W. edited and revised manuscript; P.B., I.B., J.R., E.S., B.V., J.B., B.K., G.L., and M.W. approved final version of manuscript.
